# Transcriptional Regulation of the Ufm1 Conjugation System in Response to Disturbance of the Endoplasmic Reticulum Homeostasis and Inhibition of Vesicle Trafficking

**DOI:** 10.1371/journal.pone.0048587

**Published:** 2012-11-13

**Authors:** Yinghua Zhang, Mingsheng Zhang, Jianchun Wu, Guohua Lei, Honglin Li

**Affiliations:** 1 Department of Biochemistry and Molecular Biology, Cancer Center, Georgia Health Sciences University, Augusta, Georgia, United States of America; 2 Department of Oncology, Tongji Hospital, Tongji Medical College, Huazhong University of Science and Technology, Wuhan, China; 3 Cancer Center, University of Illinois at Chicago, Chicago, Illinois, United States of America; 4 Department of Biophysics, Southern Medical University, Guangzhou, Guangdong, China; Vanderbilt University, United States of America

## Abstract

Homeostasis of the endoplasmic reticulum (ER) is essential for normal cellular functions. Disturbance of this homeostasis causes ER stress and activates the Unfolded Protein Response (UPR). The Ufm1 conjugation system is a novel Ubiquitin-like (Ubl) system whose physiological target(s) and biological functions remain largely undefined. Genetic study has demonstrated that the Ufm1-activating enzyme Uba5 is indispensible for erythroid differentiation in mice, highlighting the importance of this novel system in animal development. In this report we present the evidence for involvement of RCAD/Ufl1, a putative Ufm1-specific E3 ligase, and its binding partner C53/LZAP protein in ufmylation of endogenous Ufm1 targets. Moreover, we found that the Ufm1 system was transcriptionally up-regulated by disturbance of the ER homeostasis and inhibition of vesicle trafficking. Using luciferase reporter and ChIP assays, we dissected the Ufm1 promoter and found that Ufm1 was a potential target of Xbp-1, one of crucial transcription factors in UPR. We further examined the effect of Xbp-1 deficiency on the expression of the Ufm1 components. Interestingly, the expression of Ufm1, Uba5, RCAD/Ufl1 and C53/LZAP in wild-type mouse embryonic fibroblasts (MEFs) was significantly induced by inhibition of vesicle trafficking, but the induction was negated by Xbp-1 deficiency. Finally, we found that knockdown of the Ufm1 system in U2OS cells triggered UPR and amplification of the ER network. Taken together, our study provided critical insight into the regulatory mechanism of the Ufm1 system and established a direct link between this novel Ubl system and the ER network.

## Introduction

The endoplasmic reticulum (ER) is an organelle that plays essential roles in lipid biosynthesis, protein folding and calcium homeostasis. By adjusting the protein-folding capacity, cells maintain homeostatic control of protein influx and secretion, thereby ensuring the quality of cell-surface and secreted proteins. Perturbation of the ER homeostasis leads to ER stress and activation of the Unfolded Protein Response (UPR) [1], [2]. Generally, the UPR includes four effector responses. First, protein synthesis and translocation into the ER is attenuated, thereby reducing protein load in the ER. Second, expression of chaperone proteins and other proteins that handle unfolded proteins is elevated to increase the protein-folding capacity. Third, the capacity of ER-associated degradation (ERAD) is enhanced to clear unfolded proteins. Finally, if a homeostasis cannot be re-established, cells undergo apoptosis. At the molecular level, three apical signal transducers have been identified, including protein kinase RNA-like ER kinase (PERK), inositol-requiring protein-1 (IRE1) and activating transcription factor 6 (ATF6) [3]. IRE1 is a type I transmembrane protein that has a stress-sensing lumen domain and a cytoplasmic portion containing both a Ser/Thr kinase domain and an endonuclease domain [4], [5]. Accumulation of unfolded proteins in the ER triggers IRE1’s endonuclease activity that produces a precise cleavage of an intron from X-box-binding protein 1 (Xbp-1) mRNA to generate a potent transcriptional transactivator Xbp-1s [6]–[8]. Xbp-1s subsequently translocates into the nucleus and induces expression of the genes such as chaperones and ERAD components [7], [9]. Similar to IRE1, PERK is also a type I transmembrane protein that has a stress-sensing lumen domain and a cytoplasmic kinase domain [10]. Upon the ER stress, active PERK phosphorylates the α-subunit of eukaryotic translation initiation factor-2 (eIF2α) at ser51, which leads to attenuation of translation initiation and global reduction of protein synthesis [11]. The third transducer is a bZIP family transcription factor ATF6 that is normally tethered to ER membranes. Under ER stress, ATF6 is released from the ER and translocates to the Golgi, where it is cleaved by proteases (site 1 and site 2 proteases) [12]–[14]. The cytoplasmic portion of ATF6 is released and moves into the nucleus to activate expression of genes that are associated with protein folding and ERAD [15], [16]. Together, these cellular signaling pathways alleviate the ER stress and restore the ER homeostasis.

Ubiquitin (Ub) and Ubiquitin-like (Ubl) protein modifiers play crucial roles in many cellular processes such as gene expression, signal transduction, and cell cycle progression [17]. Human Ubiquitin-fold modifier 1 (Ufm1) is a newly identified Ubl with 85 amino acid residues [18]. Despite a very limited sequence identity (16%) with Ub, human Ufm1 displays a solution structure of ubiquitin fold with specific α-sheets and an α-helix [19]. However, the surface electrostatic potential of human Ufm1 is markedly different from those of Ub and NEDD8, and a cluster of the acidic residues in the α1 surface of Ub and NEDD8 are not present in Ufm1 [19]. Ufm1 is synthesized as a precursor and is processed by cysteine proteases, UfSP1 and UfSP2, at the C-terminus to expose the conserved Gly^83^ residue [20]. Processed Ufm1 is activated by Uba5, the Ufm1 activating enzyme, to form Ufm1-Uba5 thioester complex [18]. Activated Ufm1 is then transferred to the catalytic cysteine of Ufc1, the Ufm1 conjugating enzyme [18]. With the help of E3s, Ufm1 is presumed to modify its protein targets.

Recently, Tatsumi *et al* reported identification of KIAA0776 protein (also known as Ufl1, RCAD, NLBP, Maxer) as a novel type of E3 ligase for the Ufm1 system [21]–[24]. The study showed that Ufl1 promoted ufmylation of another novel protein C20orf116 (also known as DDRGK1, Dashurin and UFBP1) [21], [22], [25], [26], yet the functional impact of ufmylation of C20orf116 remains unclear. A recent genetic study also revealed that the Ufm1-activating enzyme Uba5 is essential for erythropoiesis, highlighting the important role of the Ufm1 system in animal development [27]. There have been reports that Ufm1 is up-regulated in type 2 diabetes and ischemic heart injury, both pathological conditions which are associated with activation of ER stress response [28], [29]. More recently, Lemaire *et al* reported that ER stress induced expression of Ufm1, its target UFBP1 and the E3 ligase Ufl1 in mouse pancreatic beta-cell line INS-1E [25]. Interestingly, knockdown of Ufm1 or its target rendered INS-1E cells sensitive to ER stress-induced apoptosis [25]. These results indicate the possible link between the Ufm1 system and the ER function. In this study we further investigated the relationship between these two systems.

## Materials and Methods

### Cell Culture

HCT116 cells were grown in McCOY’S 5A medium supplemented with 10% fetal bovine serum. HepG2 cells, 293T cells and mouse embryonic fibroblasts (MEFs) derived from Xbp-1^−/−^ embryos were cultured in Dulbecco’s modified Eagle’s medium (DMEM) supplemented with 10% fetal bovine serum. The cells were treated with thapsigargin (TG), tunicamycin (TM) or Brefeldin A (BFA) to induce ER stress conditions. Chemicals and reagents were purchased from Sigma and Calbiochem. Wild-type and Xbp-1^−/−^ MEF cell lines are a gift of Dr. Laurie H. Glimcher (Harvard School of Public Health) [9]. Wild-type and PERK knockout MEF cell lines were purchased from ATCC, and cultured in DMEM supplemented with 10% fetal bovine serum and 0.05 mM 2-Mercaptoethanol.

### Plasmids

Two expression plasmids for the Flag tagged mouse unspliced form (Xbp-1u) and spliced form of Xbp-1 (Xbp-1s) were a gift of Dr. David Ron (Addgene plasmids #21832 and 21833). Human Ufm1 promoter deletion plasmids were constructed by PCR amplification. The primers used for amplification were as follows:

−1477/Luc, 5′-GGGCTAGCTGACAATAGGTAGACAC-3′ and.

5′-GGCTCGAGGGTGGTGCCGGAATGAATCCAGAA-3′;

−595/Luc, 5′-GGGCTAGCGTTATAAATCAGTATCGG-3′ and.

5′-GGCTCGAGGGTGGTGCCGGAATGAATCCAGAA-3′;

−196/Luc, 5′-GGGCTAGCTTCTTAGATATATAACG-3′ and.

5′-GGCTCGAGGGTGGTGCCGGAATGAATCCAGAA-3′.

−1477/Luc contained the −1477 to +39 region of the human Ufm1 promoter, while −595/Luc included the −595 to +39 region. −196/Luc contained the −196 to +39 region. Each PCR product was cloned into the Nhe I/Xho I sites of pGL3 vector. Putative Xbp-1-binding site deletion plasmid −196 ΔXbp1/Luc was also constructed by two-round PCR amplification. Fragment one was amplified by primer pairs 5′-GGGCTAGCTTCTTAGATATATAACG-3′ and 5′-GCCCCGCTCCACTCACGTCTGCAATCTGGGGACCTC-3′. Fragment two was amplified by primer pairs 5′-GAGGTCCCCAGATTGCAGACGTGAGTGGAGCGGGGC-3′ and 5′- GGCTCGAGGGTGGTGCCGGAATGAATCCAGAA-3′. The two fragments were annealed and then amplified by respective primers. The PCR product was cloned into the Nhe I/Xho I sites of pGL3 vector.

### Transfection and Reporter Assays

293T cells (150×10^3^ cells in 12-well plate) were transfected with the Lipofectamine 2000 reagent according to the manufacturer’s protocol. Cells were transfected with a reporter plasmid (200 ng) carrying the firefly luciferase gene and the reference plasmid pRL-TK (20 ng) carrying the *Renilla* luciferase gene in the presence or absence of an effector protein expression plasmid (500 ng). After 24 h, the cells were treated with TG (0.5 µM) for 16 hours, and then lysed in 100 µl of Passive Lysis Buffer (Promega). The firefly and *Renilla* luciferase activities were measured using a Dual-Luciferase Reporter Assay System (Promega) and a GLOMAX™ 20/20 luminometer. The relative luciferase activity was defined as the ratio of firefly luciferase activity to *Renilla* luciferase activity. Similar reporter assay was also conducted using wild-type and Xbp-1^−/−^ MEF cells.

### siRNAs and shRNAs

Silencer Select predesigned siRNAs were purchased from Ambion, Inc. The following are the sense sequences of siRNAs we used in this study: Ufm1-5: GUUGGAAGUUGUUAAUAUC; Ufm1-7: GAACUGCGGAUUAUUCCUA. The negative control siRNAs were Silencer Select negative control 1 and 2 siRNAs that were purchased from Ambion. Reverse transfections were performed using Hiperfect (Qiagen) following the manufacturer's instruction with minor modification. 10 nM siRNA was used as the standard concentration in our knockdown assays.

The following are the sequences of lentiviral shRNAs used in this study: C53/LZAP (GCAGGAGATTATAGCTCTGTA), RCAD/Ufl1 (GCTTCTTTACTCTGTGCTTGA), Ufm1 shRNA (GTGTTGGAAGTTGTTAATATC), Uba5 shRNA (CCTCAGTGTGATGACAGAAAT), Ufc1 shRNA (GCATCACACTTAACTCATCTA), and mouse ATF6α (GCAGTCGATTATCAG CATACA). shRNA lentiviral vectors were constructed using pLKO.1 vector (Sigma). Lentiviruses were prepared using 293FT packaging cell line according to the manufacturer's instruction (Invitrogen). For knockdown experiments, cells were infected with lentiviruses expressing either scramble or other shRNAs. At 24 hours post-infection, cells were selected with puromycin (1.5 µg/ml) and cultured for extra 3–6 days. The knockdown efficiency was evaluated by immunoblotting or real-time PCR.

### Antibodies, Immunoblotting and Immunostaining

The procedures for immunoblotting and immunostaining were described previously [22]. The following antibodies at indicated dilutions were used for immunoblotting: GAPDH (Santa Cruz Biotechnology, 1∶10,000), anti-Flag M2 (Sigma, 1∶2,000), Actin (Sigma, 1∶5000), C53 rat monoclonal antibody (made by the Li lab, 1∶1,000 dilution), RCAD rat polyclonal antibody (made by the Li lab, affinity purified, 1∶500), Ufm1 rat polyclonal antibody (made by the Li lab, affinity purified, 1∶100), Ufc1 rat polyclonal antibody (made by the Li lab, affinity purified, 1∶100), Grp78 (Sigma, 1∶2000), PDI (Sigma, 1∶3000), Calnexin (Sigma, 1∶1000), CHOP (Santa Cruz, 1∶1000). Species-specific horseradish peroxidase- and fluorophore-conjugated secondary antibodies were obtained from Jackson ImmunoResearch.

### RNA Isolation and Real-time PCR

Cells (1×10^6^) were collected and washed with ice-cold phosphate-buffered saline. mRNA was purified by RNeasy mini kit (Qiagen). The first-strand cDNA was synthesized by Superscript first-strand synthesis system for RT-PCR (Invitrogen), and used as the template for real-time PCR. QuantiTect SYBR Green PCR kit (Qiagen) was used for RT-PCR assays that were run on ABI 7500 RT-PCR system and analyzed by the relevant software. The primers were used for RT-PCR:

human GAPDH:

5′-AAGGTGAAGGTCGGAGTCAA-3′ (F); 5′-CCATGTAGTTGAGGTCAATGAAGG-3′ (R); human Uba5:

5′-TGGAATCTGGGGTCAGTGAAA-3′ (F); 5′- AGCAACTACAAGTGGTGGAGC -3′ (R);

human RCAD/Ufl1:

5′-AGCAAACAGGCCTCAACTGT-3′ (F); 5′-TTTCTGGTGCATCAGCTCAC-3′ (R);

human Ufm1:

5′-CAGTGTTCCTGAAAGTACACCTT-3′ (F); 5′- CCGCAGTTCTGAACCATGTTTTA-3′ (R); human Ufc1:

5′-TGCTGACAACGATTGGTTCC-3′ (F); 5′-GGGGCAGTAGTAGGATATGTGAT-3′ (R);

Mouse GAPDH:

5′-AACTTTGGCATTGTGGAAGG-3′ (F); 5′-CAGCTTCCAACTCGGTCTCT-3′ (R);

mouse Uba5:

5′-CAAGCTATGTTCACGGCAGA-3′ (F); 5′- AGTTGTTTTGCCCACCACTC -3′ (R);

mouse RCAD/Ufl1:

5′-AGCAAACAGGCCTCAACTGT-3′ (F); 5′-TTTCTGGTGCATCAGCTCAC-3′ (R);

mouse Ufm1:

5′-CCGTTCACAGCAGTGCTAAA-3′ (F); 5′- CAGCTTCCAACTCGGTCTCT-3′ (R);

mouse Ufc1:

5′-AACTGCACTTCCGCAGTTTT-3′ (F); 5′-CTCCAGTCGGAACCAATCAT-3′ (R);

mouse beta-actin:

5′-CGACATCAGGAAGGACCTGT-3′(F); 5′-ACATCTGCTGGAAGGTGGAC-3′ (R).

### Xbp-1 Splicing Assay

Xbp-1 splicing assay was conducted to evaluate the activation of Xbp-1 in response to ER stress. RNA was isolated from the cells treated with ER stress inducers, and cDNA was synthesized and subject to PCR amplification using human Xbp-1 primers: GGAGTTAAGACAGCGCTTGG (F) and ACTGGGTCCAAGTTGTCCAG (R). Unspliced and spliced Xbp-1 mRNA species were analyzed by 2% agarose gel electrophoresis.

### Chromatin Immunoprecipitation (ChIP) Assay

*In vivo* binding of Xbp-1s to Ufm1 promoter was investigated using the ChIP assay kit (Invitrogen). Human HCT116 cells were transiently transfected with expression plasmid encoding Flag tagged Xbp-1s. Protein and DNA were cross-linked by formaldehyde treatment and lysed. DNA was sheared by sonication. Cell lysates were subjected to immunoprecipitation with control rabbit IgG or with Xbp-1 antibody (Santa Cruz, sc-7160). Purified DNA from the cell lysate and DNA recovered from immunoprecipitation were amplified by PCR. The primers for Ufm1 are 5′- GTCCCCAGCACACTAGAGGA-3′ (forward) and 5′- GGAAAAGAGCGGGAGAG AGT- 3′ (reverse). The primers for Erdj4 are 5′- GCAGCAACAACAGTTTTCCA-3′ (forward) and 5′- GCACCCTAATCTCGGTCGTA- 3′(reverse). The ChIP assays were also performed in HCT116 cells treated with TG (1 µM for 16 hours).

## Results

### 1. RCAD/Ufl1 and its Binding Protein C53/LZAP were Involved in Ufmylation of Endogenous Ufm1 Targets

**Figure 1:**
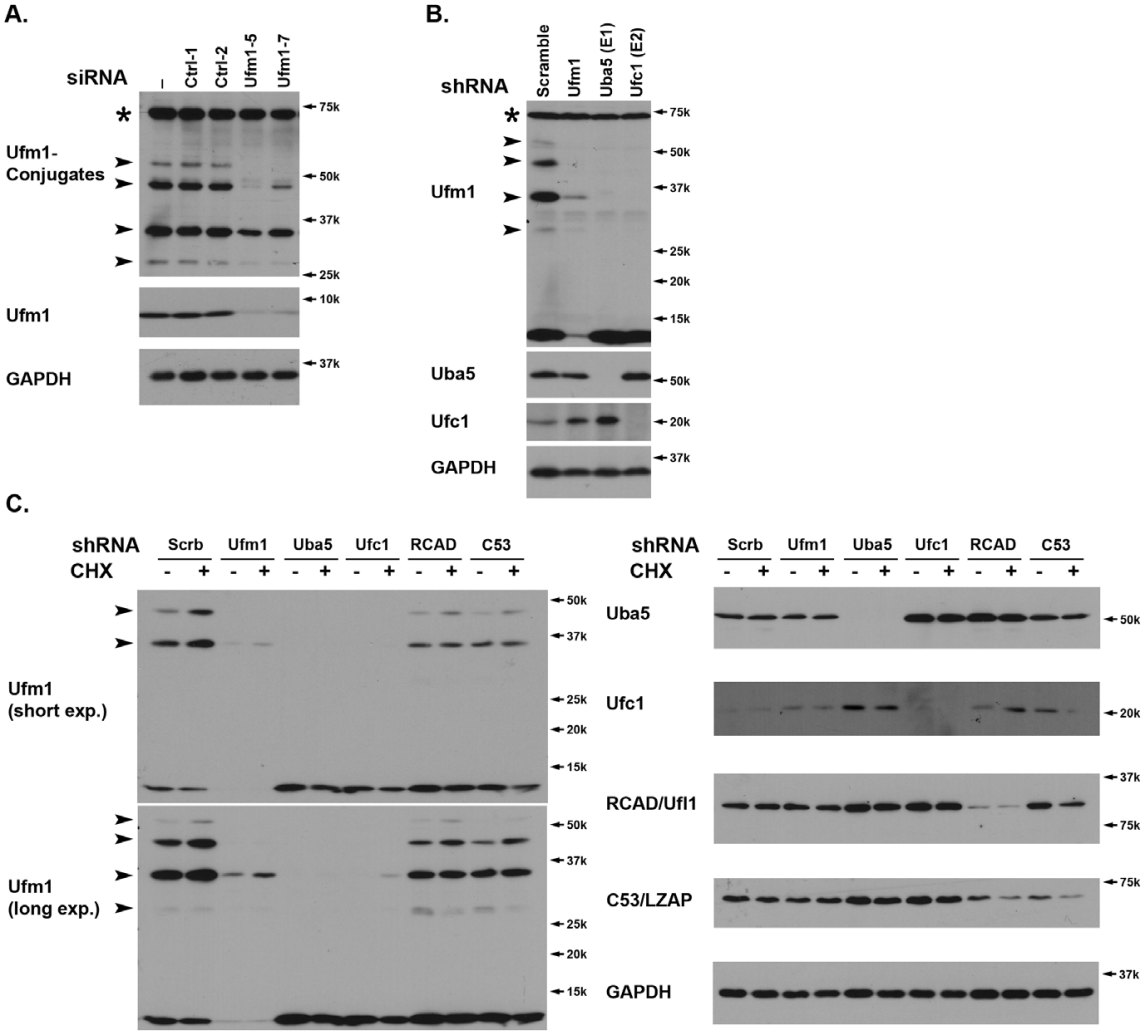
RCAD/Ufl1 and its binding partner C53/LZAP were involved in ufmylation of endogenous Ufm1 targets. **A.** The endogenous Ufm1 conjugates. HCT116 cells were transiently transfected with siRNAs, and the cell lysates were collected two days after transfection and subject to WB using Ufm1 antibody. Specific Ufm1 conjugates were marked by arrowheads, and Ufm1 knockdown efficiency was evaluated by Ufm1 immunoblotting. A “70 kD” non-specific band marked by “*”. **B.** Ufmylation of endogenous targets was reduced by shRNA-mediated knockdown of Ufm1, Uba5 and Ufc1. HCT116 cells were infected with lentiviral vectors expressing specific Ufm1, Uba5 and Ufc1 shRNAs. The cells were selected with puromycin and the cell lysates were collected after 4-day incubation. Knockdown of specific genes were confirmed by immunoblotting of specific antibodies, respectively. The Ufm1 conjugates were marked by arrowheads. **C.** RCAD/Ufl1 and its binding partner C53/LZAP were involved in ufmylation of endogenous Ufm1 targets. HCT116 cells were infected with specific lentiviral shRNAs as indicated, and knockdown of corresponding genes was confirmed by immunoblotting. Scramble shRNA was used as the negative control. After 4-day selection and incubation, the cells were treated with cycloheximide (CHX, 10 µg/ml) for 6 hours, and the cell lysates were collected and subject to immunoblotting.

Although it has been reported that multiple targets were ufmylated in tissue culture cells and animals, the identity of those targets remains largely elusive [18]. In attempt to investigate the endogenous ufmylation, we developed an affinity-purified Ufm1 antibody to detect endogenous Ufm1 conjugates. In addition to endogenous Ufm1, this antibody detected extra bands, including 28 kDa, 34 kDa, 45 kDa, 52 kDa and 70 kDa (Figure 1A) in colorectal cancer cell HCT116. To further demonstrate the specificity of our antibody and confirm if those bands are the Ufm1 conjugates, we examined the lysates of Ufm1 knockdown cells. Both Ufm1 siRNAs was able to effectively knock down endogenous Ufm1 (Figure 1A). Interestingly, except for 70 kDa band, other bands were significantly decreased in two Ufm1 knockdown cell lysates, suggesting that those bands are potential Ufm1 conjugates. We also utilized lentiviral shRNAs to knockdown endogenous Ufm1, Uba5 and Ufc1. As shown in Figure 1B, knockdown of either Uba5 or Ufc1 resulted in the reduction of Ufm1 conjugates. Interestingly, we consistently observed a slight increase of Ufc1 in the Uba5 knockdown cells (Figure 1B), indicating the existence of possible compensatory or feed-back mechanism for the Ufm1 system.

RCAD/Ufl1 is a putative E3 ligase for ufmylation of C20orf116 protein, and regulates protein stability of its binding partner C53/LZAP [21]–[23]. We tested if RCAD/Ufl1 was also involved in ufmylation of other potential targets. Protein translation inhibitor cycloheximide (CHX) enhanced ufmylation of putative Ufm1 targets, which was consistent with the previous report (Figure 1C) [25]. Intriguingly, knockdown of either RCAD/Ufl1 or C53/LZAP significantly diminished ufmylation of endogenous targets (Figure 1C). This result suggests that RCAD/Ufl1, along with its binding partner C53/LZAP, may play a general role in ufmylation of multiple targets.

### 2. The Ufm1 System was Up-regulated by ER Stresses in Multiple Cancer Cell Lines

**Figure 2:**
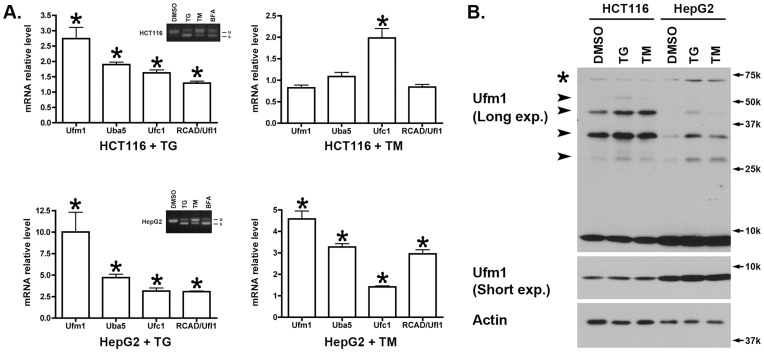
Expression of the Ufm1 system was induced by ER stress. **A.** RT-PCR results of HCT116 and HepG2 cells treated with TG (1 µM for 16 hours) and TM (10 µM for 16 hours). The results represented mean ± SEM, p value <0.01 (marked by *). The inserts in Fig. 2A showed the results of Xbp-1 splicing assays. “u” indicated the unspliced form of Xbp-1, while “s” is the spliced form. **B.** Immunoblotting of the cell lysates of HCT116 and HepG2 treated with TG and TM. Specific Ufm1 conjugates were marked by arrowheads, and the non-specific 70 kD band was indicated by a star. “Long exp.” was long exposure of the blot, while “Short exp.” was short exposure of the blot in Enhanced Chemiluminescence (ECL).

It has been reported that certain components of the Ufm1 conjugation system are up-regulated in animal models and tissue culture cells under ER stresses [25], [28], [29]. We further examined whether the expression of its components was affected by ER stress. As shown in Figure 2A, Ufm1 was induced more than two fold in HCT116 cells in response to treatment of thapsgargin (TG), a potent inhibitor of ER Ca^2+^ ATPase and strong inducer of ER stress. Interestingly, other components of the Ufm1 system, including Uba5, Ufc1 and RCAD/Ufl1, were also up-regulated to various degrees (Figure 2A). We also examined the effect of ER stress inducer tunicamycin (TM), an inhibitor of glycosylation. Tunicamycin had a modest effect on mRNA levels of the Ufm1 system in HCT116 cells (Figure 2A). To confirm the induction of UPR in HCT116 cells treated with TG or TM, we examined Xbp-1 splicing, a marker for Xbp-1 activation and UPR. TG appeared more potent to induce Xbp-1 splicing in HCT116 than TM (Figure 2A). In comparison to HCT116, both TG and TM exerted more prominent effect on the expression of the Ufm1 system In HepG2 cells (Figure 2A). In addition to examining mRNA level, we also looked at the protein level of the Ufm1 system. Although inhibition of protein synthesis by CHX led to reduction of protein levels of Ufm1 and other components (Figure 1C), Ufm1 protein slightly increased in TG and TM-treated cells (Figure 2B), suggesting that Ufm1 is still translated under ER stress conditions in which global protein synthesis is generally suppressed. Moreover, the putative Ufm1 conjugation was also elevated under ER stresses (Figure 2B). Taken together, these data suggest that acute ER stress leads to up-regulation of the Ufm1 system.

### 3. Ufm1 is a Potential Target of Xbp-1

**Figure 3:**
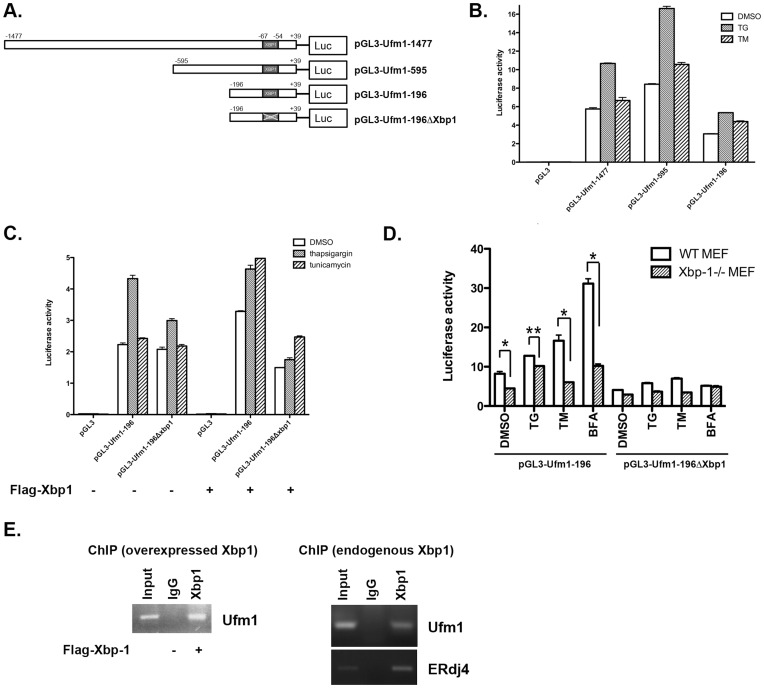
Ufm1 is a potential target of Xbp-1. **A.** The constructs of the Ufm1 promoter used for luciferase reporter assays. Human Ufm1 promoter sequence was amplified from the genome of HCT116 cells. **B.** The minimal Ufm1 promoter responded to ER stresses. Various Ufm1 promoter constructs were transfected into 293T cells that were subsequently treated with TG (0.5 µM) and TM (10 µM) for 24 hours. The promoter activity was measured by dual luciferase reporter assays (Promega). **C.** The putative Xbp-1 binding site was responsible for Xbp-1-mediated induction of Ufm1. 293T cells were transfected with indicated Ufm1 promoter constructs along with Xbp-1 expression vector. The cells were subsequently treated with TG and TM, and the promoter activity was measured by dual luciferase assays. **D.** The Ufm1 promoter activity in wild-type and Xbp-1^−/−^ MEFs. The Ufm1 protomer reporter was transfected into wild-type and Xbp-1^−/−^ MEFs, and the promoter activity was measured by dual luciferase reporter assays (Promega). The results represented mean ± SD. *p value <0.01 and **p value <0.05. **E.** CHIP assay. The Xbp-1-DNA complex was immunoprecipitated with Xbp-1 antibody, and subject to PCR using the primers specific for Ufm1 and ERdj4 promoters.

To study the transcriptional regulation of Ufm1 expression, we isolated a 1.5 kb genomic fragment upstream of the putative Ufm1 transcription start site and identified the potential transcription factor binding sites. As analyzed by an online tool cisRED [30], the Ufm1 promoter contains many cis-elements for potential transcription factors such as E2F-1, AP-2 and HIF-1. Interestingly, the sequence between −67 to −54 (AGGGAGCCGTGGA) contains a conserved binding site for Xbp-1, a potent transcription factor that plays a critical role in UPR [2]. We performed luciferase report assays to test if Ufm1 is a target of Xbp-1. The 1.5 kb promoter and its deletion constructs were subcloned into pGL3 vector (Figure 3A). As shown in Figure 3B, the minimal promoter (Ufm1-196) was able to initiate the transcription of the Ufm1 promoter even though with less efficiency. The promoter activity increased significantly when the cells were treated with TG (Figure 3B). To determine if the putative Xbp-1 binding site was responsible for the increase of promoter activity, we deleted the putative Xbp-1 site and the resulting promote (Ufm1-196ΔXbp1) was not activated well by either TG treatment or overexpression of Xbp-1 protein (Figure 3C). Furthermore, we performed the reporter assay in Xbp-1 deficient MEF cells. Apparently, the minimal promoter (Ufm1-196) responded well to ER stress inducers in wild-type MEF cells, but its response was significantly suppressed in Xbp-1^−/−^ MEFs (Figure 3D). Finally, we performed ChIP assay to test if the Ufm1 gene is a direct target of Xbp-1. ERdj4, a known Xbp-1 target, was used as the positive control. As shown in Figure 3E, the PCR product of the Ufm1 promoter region was present in the immunoprecipitates of either overexpressed or endogenous Xbp-1s. Together, our results suggest that Ufm1 is a potential target of Xbp-1.

### 4. The Effect of the UPR Pathways on the Expression of the Ufm1 System in MEFs

**Figure 4:**
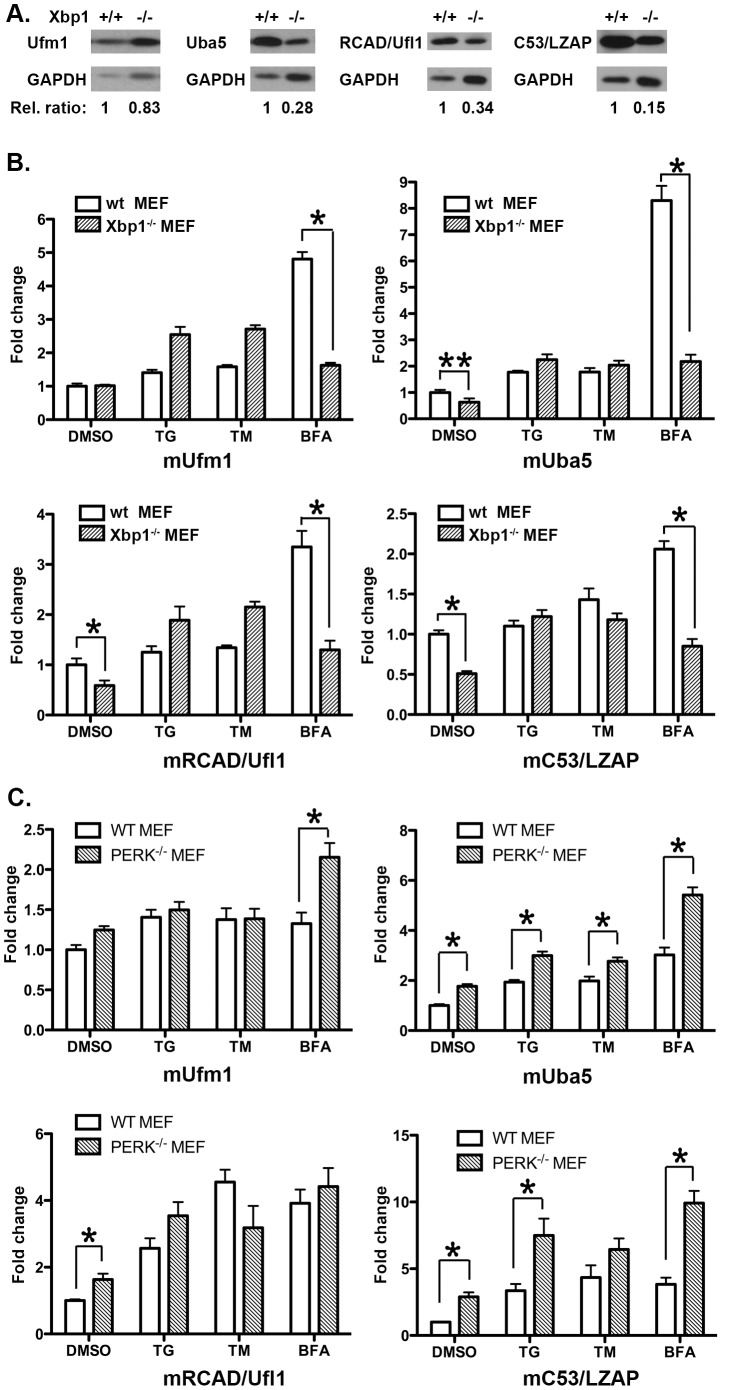
The effect of the UPR pathways on the expression of the Ufm1 system. **A.** Immunoblotting of MEF cell lysates using Ufm1, Uba5, RCAD/Ufl1 and C53/LZAP antibodies. GAPDH was used as a loading control. Relative ratios of proteins were measured against GAPDH using Image J software. **B.** The mRNA levels of Ufm1, Uba5, RCAD/Ufl1 and C53/LZAP in wild-type and Xbp-1^−/−^ MEF cells that were treated with ER stress-inducing agents (TG, 0.5 µM for 16 hour; TM, 10 µM for 16 hours; and BFA, 0.5 µg/ml for 16 hours). **C.** The mRNA levels of Ufm1, Uba5, RCAD/Ufl1 and C53/LZAP in wild-type and PERK^−/−^ MEF cells that were treated with ER stress-inducing agents. The results represented mean ± SD. *p value <0.01.

Except for the Ufm1 promoter, sequence search failed to reveal any obvious Xbp-1 binding sites in the promoters of Uba5, RCAD/Ufl1 and C53/LZAP (data not shown). To further investigate if Xbp-1 is involved in regulating expression of the Ufm1 system, we examined the protein level of various components in Xbp-1 deficient MEF cells. Under normal culture condition, the protein level of Ufm1 in Xbp-1^−/−^ MEFs was relatively similar to the one in wild-type MEF. However, the protein level of Uba5, RCAD/Ufl1 and C53/LZAP was significantly lower in Xbp-1^−/−^ MEFs (Figure 4A). Accordingly, the mRNA level of Uba5, RCAD/Ufl1 and C53/LZAP was also lower in Xbp-1^−/−^ MEF cells, while Ufm1 remained comparable in wild-type and knockout cells (Figure 4B). We further examined their expression in MEF cells treated with various ER stress inducers. Treatment of TG and TM modestly raised the expression of the Ufm1 components. The induction was not reduced by Xbp-1 deficiency (Figure 4B), and the expression of Ufm1 and RCAD/Ufl1 was even slightly higher in Xbp-1^−/−^ MEFs. In contrast, Ufm1, Uba5, RCAD/Ufl1 and C53/LZAP were significantly up-regulated in wild-type MEF cells treated with Brefeldin A, a potent inhibitor of vesicle trafficking, and the induction was negated by Xbp-1 deficiency (Figure 4B). As a negative control, beta-actin expression was not significantly changed by TG, TM or BFA treatment (Figure S1a).

To further analyze the contribution of other branches of the UPR to the regulation of the Ufm1 system, we first attempted to examine the effect of PERK pathway using PERK−/− MEFs. As shown in Figure 4C, expression of Ufm1, Uba5, RCAD/Ufl1 and C53/LZAP was up-regulated by the treatment of TG, TM and BFA in wild-type MEFs, which was consistent with the results described above. Unlike Xbp-1 deficiency, however, PERK deficiency did not suppress BFA-induced up-regulation of the Ufm1 system (Figure 4C). Interestingly, PERK deficiency appeared to generally enhance the expression of certain Ufm1 components (Figure 4C). In addition, we tested the effect of the ATF6α branch using ATF6α knockdown MEF cells. ATF6α shRNA resulted in 60% knockdown of endogenous protein (data not shown), and knockdown of ATF6α did not suppress the up-regulation of the Ufm1 system induced by BFA (Figure S1b). Taken together, our results provided more evidence for transcriptional regulation of the Ufm1 system in response to ER stress. Additionally, our data suggest a key role of Xbp-1 in up-regulation of the Ufm1 system induced by inhibition of vesicle trafficking.

### 5. Knockdown of the Ufm1 System Resulted in UPR Activation

**Figure 5:**
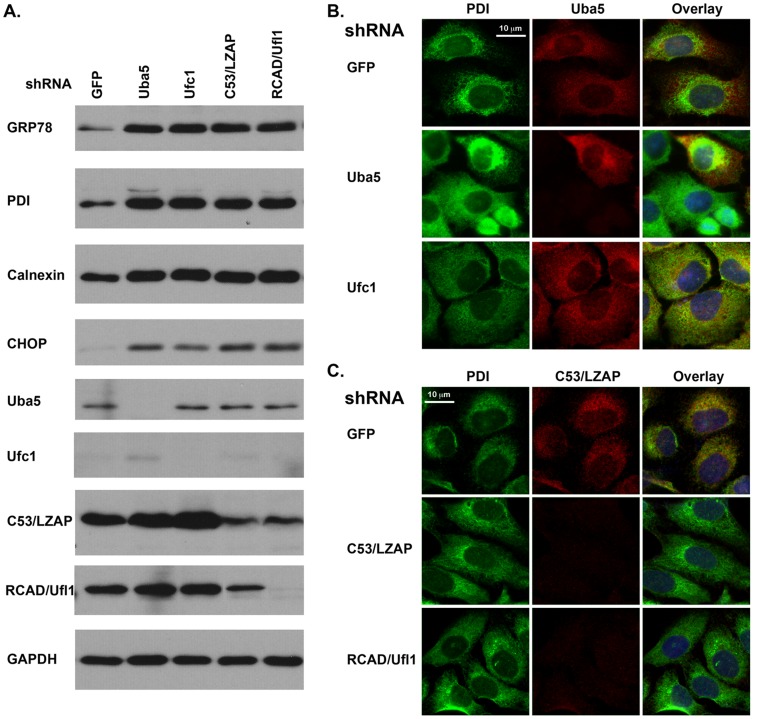
Knockdown of the Ufm1 system resulted in activation of UPR. **A.** Up-regulation of ER chaperone proteins and CHOP in U2OS cells with knockdown of the Ufm1 system. U2OS cells were infected with lentiviral shRNAs, selected with puromycin (1.5 µg/ml). Cell lysates were collected after 6-day incubation and subject to immunoblotting with indicated antibodies. Knockdown of the Ufm1 components were confirmed by immunoblotting. **B.** Immunostaining of PDI in Uba5 and Ufc1 knockdown cells. C. Immunostaining of PDI in C53/LZAP and RCAD/Ufl1 knockdown cells. U2OS cells were subjected to immunostaining of PDI,. Knockdown of Uba5 was confirmed by Uba5 staining, while knockdown of RCAD/Ufl1 and C53/LZAP was confirmed by C53 staining. The images were acquired by Zeiss Axio Observer D1 and Axiovision software.

Despite the fact that RCAD/Ufl1 and its target C20orf116 are the ER proteins, it is not clear if they are involved in normal ER function [21], [22]. We examined the impact of knockdown of the Ufm1 system on the ER in U2OS cells. Knockdown of Uba5, Ufc1, RCAD/Ufl1 and C53/LZAP slightly slowed cell proliferation of U2OS cells (data not shown). Interestingly, expression of Grp78/Bip, PDI (protein disulfide isomerase), Calnexin and CHOP/GADD153 (C/EBP homology protein) were significantly increased in the knockdown cells, indicating the activation of UPR (Figure 5A). We further examined the ER network in those cells using PDI staining. The ER network usually follows the microtubule (MT) cytoskeleton and enriches in MT-rich areas such as the centrosome and perinuclear regions. In control U2OS cells, the ER network was unevenly distributed in the cytosol and concentrated around the nuclear (Figure 5B). In contrast, knockdown of Uba5 and Ufc1 lead to the increase of PDI staining, and the ER network was more evenly distributed to the periphery, suggesting an amplification of the ER network. Similar change was also observed in RCAD/Ufl1 and C53/LZAP knockdown cells (Figure 5C). These results showed that knockdown of the Ufm1 system triggered the UPR in U2OS cells, suggesting an important role of the Ufm1 system in the normal ER functions.

## Discussion

The Ufm1 conjugation system is a novel Ubl system that shares the common features with other Ubl systems, but its physiological target(s) and cellular function remains largely undefined [18]. Recent study by Tatsumi *et al* has demonstrated that the Ufm1-activating enzyme Uba5 is indispensible for erythroid differentiation in mice [27], highlighting the importance of this novel Ubl system in animal development. In this report we showed the evidence for possible involvement of RCAD/Ufl1 and C53/LZAP proteins in ufmylation of endogenous Ufm1 targets, supporting RCAD/Ufl1’s role as a Ufm1-specific E3 ligase. Moreover, we found that the Ufm1 system was transcriptionally up-regulated in response to ER stress and inhibition of vesicle trafficking. Using luciferase reporter and CHIP assays, we demonstrated that Ufm1 was a potential target of Xbp-1, a crucial transcription factor for cellular response to ER stress. Furthermore, we examined the effect of various UPR pathways on the expression of the Ufm1 components. Interestingly, the expression of the Ufm1 system was significantly induced by BFA, a potent inhibitor of vesicle trafficking, and the induction was negated by Xbp-1 deficiency. In contrast, neither PERK deficiency nor ATF6α knockdown in MEFs had a significant effect on BFA-induced up-regulation. Finally, we found that knockdown of the Ufm1 system in U2OS cells triggered UPR and amplification of the ER network. In this study, we established a direct link between the Ufm1 conjugation system and the ER network, which may serve as a key for our better understanding of the biological function of this novel Ubl system.

Although it was reported that Ufl1 promoted ufmylation of C20orf116 *in vitro* and in an overexpression experimental system, the actual evidence for ufmylation of endogenous C20orf116 was elusive [21]. In this study, we took advantage of a Ufm1-specific antibody and potent shRNAs to demonstrate that RCAD/Ufl1 and its binding partner were indeed involved in ufmylation of endogenous Ufm1 targets (Figure 1). This result also raises an interesting possibility that RCAD/Ufl1 may function as a Ufm1-specific E3 ligase for multiple targets. Furthermore, C53/LZAP protein that interacts with both Ufm1 and RCAD/Ufl1 may also play a critical role in ufmylation of those targets even though C53/LZAP did not promote ufmylation of C20orf116 *in vitro* (our unpublished data).

Xbp-1 is a member of CREB/ATF transcription factor family and one of the most important downstream effectors in the UPR [2], [31]. Knockout mice studies have shown that it is essential for many important developmental events such as cardiac myogenesis, hepatogenesis, plasma cell differentiation and development of secretory tissues [32]–[35]. Genome-wide studies have shown that Xbp-1 controls diverse cell type- and condition-specific transcriptional regulatory networks [36]. In the UPR, the IRE1α/Xbp-1 branch is responsible for induction of a subset of ER chaperone genes [7], [9]. In this study, we used luciferase reporter and ChIP assays to identify Ufm1 as a potential direct target of Xbp-1 (Figure 3). Nonetheless, unlike other typical ER stress-induced proteins such as Grp78, Ufm1 is expressed in a relatively high level under normal condition, suggesting that its expression is not solely dependent on Xbp-1. Moreover, in Xbp-1 deficient MEFs, Ufm1 was still induced by ER stressors like TG and TM (Figure 4B). It has been known that many UPR target genes are not affected by Xbp-1 deficiency [9]. Moreover, Xbp-1 controls many transcriptional networks in a cell type and condition-specific manner [36]. Therefore, it is possible that Xbp-1-dependent regulation of Ufm1 expression may be specific for certain cell types or developmental stages, and more study using Xbp-1 knockout animals will be surely needed to test this possibility.

Another interesting observation is that the protein and mRNA levels of Uba5, RCAD/Ufl1 and C53/LZAP were much lower in Xbp-1^−/−^ MEFs even in the absence of ER stress (Figure 4). IRE1, the upstream activator of Xbp-1, can function in a process termed regulated IRE1-dependent decay (RIDD) to induce the degradation of a subset of mRNAs encoding for secreted cargo proteins and ER-resident proteins that handle folding and trafficking of cargo proteins [37]–[39]. Genetic ablation of Xbp-1 results in constitutive IRE1α activation in the liver, thereby leading to RIDD of Cyp1a2 and Cyp2e1 mRNAs [40], [41]. Whether Uba5 and other Ufm1 components are the targets of RIDD will be subject to further study.

The complexity of transcriptional regulation of the Ufm1 system is also reflected by several inconsistencies in our study, such as variations on the induction of certain Ufm1 components in response to different ER stressors. All Ufm1 components are expressed under normal cell culture conditions and mostly up-regulated by ER stress (TG and TM) and inhibition of vesicle trafficking (BFA) (Figures 2 and 4). However, the degree of induction appeared to vary, depending upon the types of cell and stressor. It has been known that susceptibility to different ER stressors differs dramatically among different cell types, yet the underlying mechanism remains largely unclear. Isolation and characterization of the promoters of individual Ufm1 components will be crucial for further understanding of their response to ER stress and relationship to the UPR pathways. In comparison to TG and TM, BFA was consistently observed as a better inducer for Ufm1 expression, and BFA-induced up-regulation of the Ufm1 components was significantly negated by Xbp-1 deficiency (Figure 4B). BFA is a potent inhibitor of certain ARF-GEFs that effectively blocks ER-to-Golgi and intra-Golgi trafficking, thereby resulting in ER stress and activation of UPR. However, BFA may activate, in addition to the UPR, other transcriptional networks that contribute to its robust effect on the Ufm1 system. Systematic expression profiling will be conducted to search for BFA-specific transcriptional activators that are responsible for its effect on the Ufm1 system.

So what are the cellular and biological functions of the Ufm1 system? Ufm1 expression was elevated in the animal models of type 2 diabetes and ischemic heart injury that are associated with ER stress response [28], [29]. Lemaire *et al* reported that Ufm1 and its putative target UFBP1/C20orf116 protected pancreatic beta cells from ER stress-induced apoptosis [25]. Knockdown of Ufm1 and UFBP1/C20orf116 rendered INS-1E cells susceptible to ER stress-induced apoptosis, but did not affect glucose stimulated insulin secretion and provoke UPR [25]. Therefore, one possible function of the Ufm1 system is to alleviate the ER stress under pathological conditions. However, Ufm1 and its components are also ubiquitously expressed in many tissues and cells under physiological conditions [22], and Uba5 is essential for animal development [27] which strongly suggests a crucial role of the Ufm1 system in normal cellular functions. We postulate that the Ufm1 system plays an important role in maintaining the ER homeostasis, and the deficiency of this system may lead to disturbance of the homeostasis and UPR activation. Interestingly, we found that knockdown of the Ufm1 components in U2OS cells triggered UPR activation and amplification of the ER network. This result was slightly different from the study reported by Lemaire *et al* [25], in which no UPR was observed in INS-1 cells with knockdown of the Ufm1 system. This discrepancy may be attributed to the difference between the cell lines and/or siRNA-mediated knockdown efficiency. Consistent with our hypothesis, the putative Ufm1 conjugates are mostly found in the endomembrane system ([21] and our unpublished observation). Both RCAD/Ufl1 and C53/LZAP are largely ER-associated proteins [21], [22]. Interestingly enough, Pfam search revealed that C53/LZAP orthologues in certain species of ants and bees consist of fusion of the C53 domain (DUF773) and Emp_gp25L domain, a conserved domain that binds to COPI and COPII complexes and plays a critical role in selective transport processes at the ER and Golgi interface (our unpublished observation). Together, these observations suggest an interesting working mechanism in which the Ufm1 system plays a critical role in vesicle trafficking, and its inhibition may lead to protein overload in the ER and UPR activation. This hypothesis will be rigorously tested by our future genetic and cellular studies.

## Supporting Information

Figure S1 A. The mRNA level of beta-actin in wild-type and Xbp-1^−/−^ MEF cells that were treated with ER stressors. B. The mRNA levels of Ufm1, Uba5, RCAD/Ufl1 and C53/LZAP in wild-type and ATF6α knockdown MEF cells that were treated with BFA.
(TIF)
