# Supplementary figures and images for: Transcriptional Regulation of the Ufm1 Conjugation System in Response to Disturbance of the Endoplasmic Reticulum Homeostasis and Inhibition of Vesicle Trafficking
Source: PLoS One. 2012 Nov 13;7(11):e48587. doi: 10.1371/journal.pone.0048587 (PMC3496721; doi:10.1371/journal.pone.0048587)

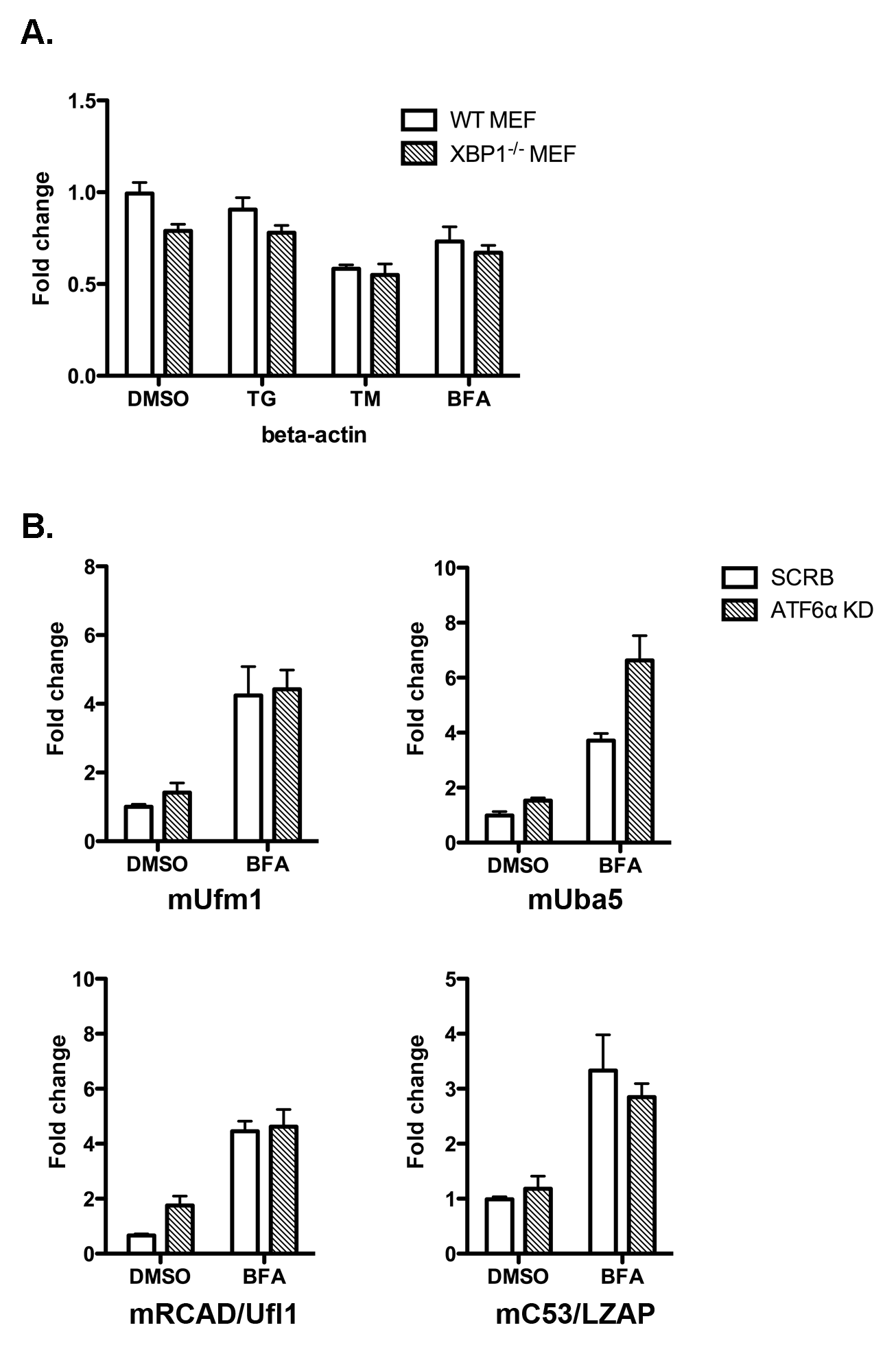

Supplement: Figure S1 — A. The mRNA level of beta-actin in wild-type and Xbp-1−/− MEF cells that were treated with ER stressors. B. The mRNA levels of Ufm1, Uba5, RCAD/Ufl1 and C53/LZAP in wild-type and ATF6α knockdown MEF cells that were treated with BFA. (TIF) [file pone.0048587.s001.tif]
